# Mu heavy chain disease with *MYD88* L265P mutation: an unusual manifestation of lymphoplasmacytic lymphoma

**DOI:** 10.1186/s13000-022-01244-1

**Published:** 2022-08-05

**Authors:** Vandana Baloda, Sarah E. Wheeler, David L. Murray, Mindy C. Kohlhagen, Jeffrey A. VosUPMC, Svetlana A. Yatsenko, Mounzer E. Agha, Miroslav Djokic, Steven H. Swerdlow, Nathanael G. Bailey

**Affiliations:** 1 Department of Pathology, UPMC, Pittsburgh, PA USA; 2 Department of Pathology, University of Pittsburgh and UPMC, Pittsburgh, PA USA; 3 Department of Laboratory Medicine and Pathology, Mayo Clinic, Rochester, MN USA; 4 Department of Pathology, Anatomy and Laboratory Medicine, West Virginia University, Morgantown, WV USA; 5 Hillman Cancer Center, University of Pittsburgh and UPMC, Pittsburgh, PA USA

## Abstract

**Background:**

Mu heavy chain disease is a rare lymphoid neoplasm characterized by vacuolated bone marrow plasma cells and secretion of defective mu immunoglobulin heavy chains. The biological basis of mu heavy chain disease is poorly understood.

**Case presentation:**

We report a case of mu heavy chain disease with *MYD88* L265P mutation and deletion of 6q, genetic aberrations that are both strongly associated with lymphoplasmacytic lymphoma/Waldenström macroglobulinemia. Identification of the truncated mu immunoglobulin was facilitated by mass spectrometric analysis of the patient’s serum.

**Conclusions:**

Mu heavy chain disease has been described as similar to chronic lymphocytic leukemia; however, the frequency of lymphocytosis in mu heavy chain disease has not been previously reported. We reviewed all previously published mu heavy chain disease reports and found that lymphocytosis is uncommon in the entity. This finding, along with the emerging genetic feature of recurrent *MYD88* mutation in mu heavy chain disease, argues that at least a significant subset of cases are more similar to lymphoplasmacytic lymphoma than to chronic lymphocytic leukemia.

## Background

Mu heavy chain disease (mu-HCD) is an exceptionally rare entity described as resembling chronic lymphocytic leukemia (CLL), with vacuolated plasma cells and secretion of a defective mu heavy chain [1]. We report a case of mu-HCD with *MYD88* L265P mutation along with deletion of 6q, supporting a biologic relationship between mu-HCD and lymphoplasmacytic lymphoma (LPL), a lymphoma that most often presents as Waldenström macroglobulinemia (WM).

## Case presentation

**Fig. 1:**
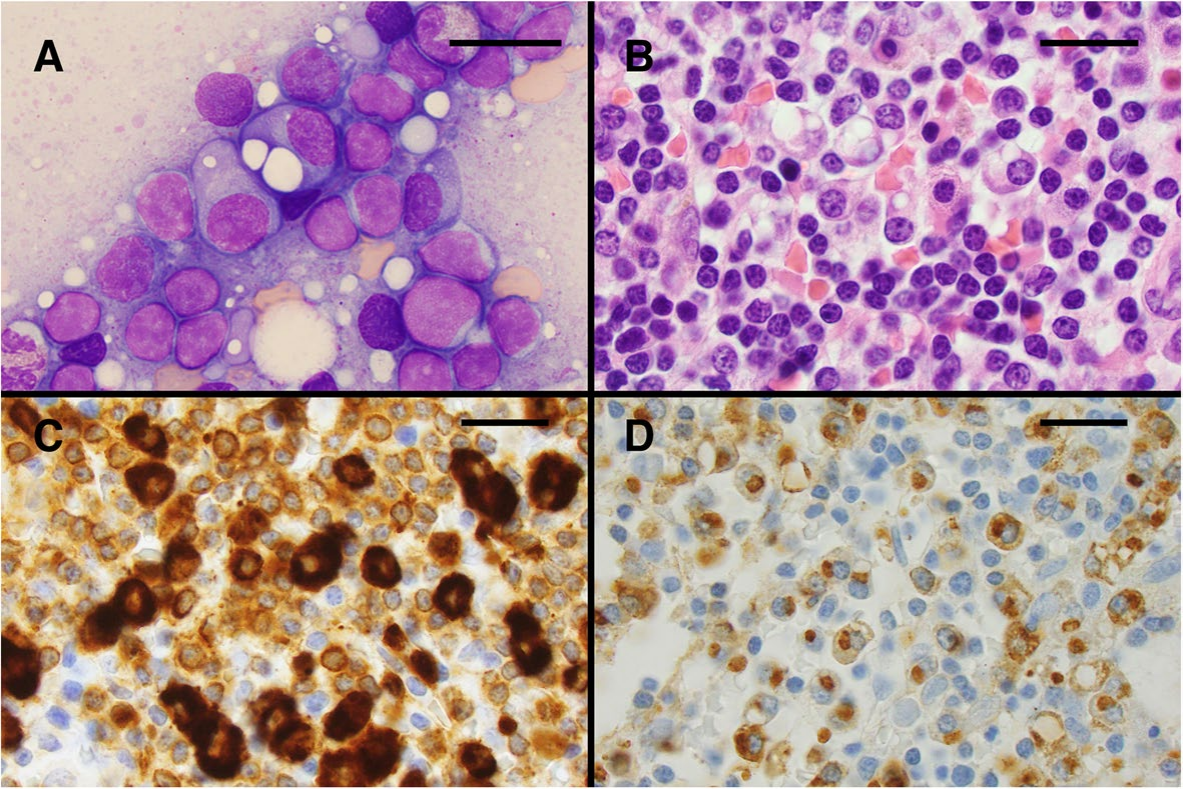
Morphologic features of mu heavy chain disease. The bone marrow was markedly hypercellular due to a diffuse proliferation of small lymphocytes and plasma cells with prominent cytoplasmic vacuolization (**A**: bone marrow aspirate, Wright-Giemsa stain, 1000 × and **B**: bone marrow core biopsy, hematoxylin and eosin stain, 1000 ×). The immunohistochemical studies performed on the bone marrow biopsy demonstrated that the B-cells and plasma cells were strongly positive for IgM (**C**: 1000 ×) and the plasmacytic component strongly expressed kappa light chains (**D**: 1000 ×). Scale bars indicate 20 µm

**Fig. 2:**
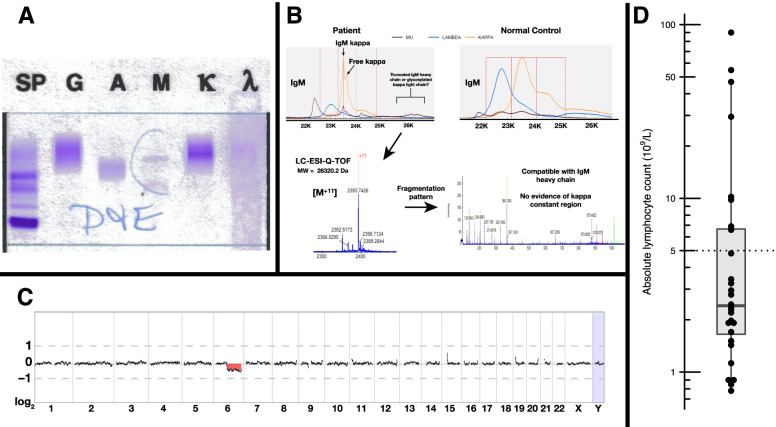
Serum protein and genetic characterization of the case and lymphocyte counts in reported cases of mu heavy chain disease. **A** Serum immunoelectrophoresis identified a monoclonal protein composed of kappa light chains with a possible faint band at IgM. **B** The serum immunoglobulin-enriched matrix-assisted laser desorption ionization time-of-flight mass spectrometry (MASS-FIX) and monoclonal immunoglobulin rapid accurate mass measurement IgM spectra for the patient demonstrating a 26,320 Da fragment of IgM heavy chain. Upper left panel: MASS-FIX IgM spectra demonstrating 3 M-proteins (An IgM kappa plus free kappa plus a suspected IgM heavy chain). Upper right panel: MASS-FIX IgM spectra for a healthy donor. Lower left panel: An LC-ESI-Q-TOF of the suspected IgM fragment for the + 11 charge state. Lower right panel: Fragmentation spectra of the suspected IgM heavy chain, demonstrating a pattern consistent with an IgM heavy chain. **C** Array comparative genomic hybridization revealed a 6q deletion. **D** Absolute lymphocyte count of mu heavy chain disease cases previously reported in the literature and including this case, plotted on a log scale. Dashed line indicates 5 × 10^9^/L threshold for lymphocytosis. Abbreviations: LC-ESI-Q-TOF: liquid chromatography coupled with electrospray ionization quadrupole time of flight mass spectrometry

A 68-year-old female presented with fatigue, dyspnea, weight loss, and anemia (8.8 g/dL). The patient had an absolute lymphocyte count (ALC) of 2.2 × 10^9^/L. A PET/CT scan did not demonstrate any lytic bone lesions or enlarged lymph nodes; the spleen size was reported as at the upper limit of normal. A bone marrow biopsy and aspirate were obtained. The bone marrow was markedly hypercellular due to a diffuse proliferation of small lymphocytes and plasma cells with prominent cytoplasmic vacuolization (Fig. 1 A and B). The B cells and plasma cells were positive for IgM by immunohistochemistry (Fig. 1C), and the plasmacytic component expressed kappa light chains (Fig. 1D). A CD5-negative, CD10-negative, kappa-restricted B-cell population was detected by flow cytometry. Serum and urine protein electrophoresis, serum free light chain testing, and serum immunoglobulin-enriched matrix-assisted laser desorption ionization time-of-flight mass spectrometry (MASS-FIX) [2] were performed. The patient had markedly elevated free kappa light chains in the serum (13,285 mg/L, reference range 3.3–19.4 mg/L), along with kappa light chains in the urine, without a significant serologically detectable heavy chain (Fig. 2A). MASS-FIX identified an abnormally small mu heavy chain (26.3 kDa) in the serum that was further characterized using liquid chromatography coupled with electrospray ionization quadrupole time of flight mass spectrometry (LC-ESI-Q-TOF) [3] (Fig. 2B). Mutational studies performed on the peripheral blood demonstrated a *MYD88* L265P mutation, and array comparative genomic hybridization performed on the bone marrow revealed a 6q deletion (Fig. 2C). The patient had normal renal function despite the Bence-Jones proteinuria. She was treated with 3 cycles of single-agent rituximab with normalization of hemoglobin and improvement of the white blood cell count.

## Discussion and conclusions

This case of mu-HCD with classic morphologic, phenotypic, and serologic features demonstrates genetic similarity to LPL with a *MYD88* L265P mutation and deletion of 6q, both common abnormalities in LPL [4, 5]. Vergneault and colleagues recently reported two cases of mu-HCD with *MYD88* L265P mutations, although vacuolated plasma cells were described in only one case and none were illustrated [6]. Detailed analysis of the mu heavy chain was also not provided. These cases, however, in addition to ours, establish that *MYD88* mutations are recurrent in mu-HCD, suggesting a relationship with LPL. The generalizability of this finding is difficult to assess as mu-HCD is extraordinarily rare, with only 30–40 cases previously reported; however, additional indirect evidence suggests that a relationship with LPL is plausible in many mu-HCD cases.

The first patient described with mu-HCD had a high ALC of approximately 50 × 10^9^/L, and the patient was clinically diagnosed with CLL [7, 8]. In 1992, Wahner-Roedler and Kyle published a summary of the then-extant literature on mu-HCD, describing the clinicopathologic features of 28 affected patients in what remains the most comprehensive description of the disease [9]. In their review, 9 of the 28 patients were reported as having been diagnosed with CLL. The 2017 WHO Classification states that mu-HCD is “a B-cell neoplasm resembling CLL” [1]; however, mu heavy chain secretion and plasmacytic differentiation are not typical features of CLL. Lymphocytosis is the *sine qua non* of CLL, but the frequency of lymphocytosis in mu-HCD is not established, as it has not been summarized in previous reviews of the entity. We attempted to review all prior reports of mu-HCD, collecting peripheral blood white blood cell and differential count information from 29 previously reported patients with mu-HCD [6, 8–29]. Including our case, ~ 73% (22 of 30) of patients with mu-HCD for whom information was available had no evidence of lymphocytosis (defined as ALC > 5 × 10^9^/L), and only 5 reported patients have had ALC greater than 10 × 10^9^/L (Fig. 2D), a much lower frequency of lymphocytosis than would be expected for a cohort of CLL patients.

Nearly all cases of CLL are positive for CD5, but the immunophenotypic characteristics of mu-HCD are not well established, as many of the reports of mu-HCD predate comprehensive immunophenotyping of lymphoid neoplasia and the recognition of the CD5 antigen on CLL cells [30]. However, at least one previously reported patient with mu-HCD with lymphocytosis (ALC ~ 29 × 10^9^/L) was negative for CD5 [14], as was our case and one of the two cases reported by Vergneault and colleagues [6]. CD5 may be expressed on LPL [31], and so reports of CD5 expression in some mu-HCD cases do not preclude classification as LPL [6].

These features suggest that most previously reported cases of mu-HCD are unlike CLL as currently understood. Our case and those of Vergneault and colleagues [6] establish a biologic relationship with LPL in at least some mu-HCD cases, which is also congruent with the presence of plasmacytic differentiation and production of IgM heavy chains in both entities. Some clinical features such as hepatosplenomegaly appear to be more common in mu-HCD than in LPL. It may be that conventional LPL associated with WM tends to manifest earlier in the course of disease than does mu-HCD due to increased pathogenicity of intact IgM paraproteins versus the truncated IgM proteins produced by mu-HCD. It remains possible that mu-HCD is a biologically heterogeneous entity, and some cases may have CLL-like genetics.

This report additionally underscores the value of novel techniques such as MASS-FIX in the detection of abnormal serum proteins. mu-HCD may be underrecognized, and patients with increased free serum light chains or Bence-Jones proteinuria without a corresponding serum heavy chain should be considered for further assessment using methods such as MASS-FIX to better evaluate for abnormal proteins that could be missed using conventional serologic techniques. Discrimination between a B-cell neoplasm with plasmacytic differentiation and a plasma cell neoplasm is highly clinically relevant, and accurate M-protein characterization is a critical component of the diagnosis. Our findings suggest that mu-HCD cases should be screened for *MYD88* mutation and establish a rationale for potential LPL-directed management of this rare entity.

## Abbreviations

mu-HCD: Mu heavy chain disease
CLL: Chronic lymphocytic leukemia
LPL: Lymphoplasmacytic lymphoma
WM: Waldenström macroglobulinemia
ALC: Absolute lymphocyte count
MASS-FIX: Serum immunoglobulin-enriched matrix-assisted laser desorption ionization time-of-flight mass spectrometry
LC-ESI-Q-TOF: Liquid chromatography coupled with electrospray ionization quadrupole time of flight mass spectrometry
