# Correction: Mu heavy chain disease with *MYD88* L265P mutation: An unusual manifestation of lymphoplasmacytic lymphoma

**DOI:** 10.1186/s13000-022-01265-w

**Published:** 2022-10-20

**Authors:** Vandana Baloda, Sarah E. Wheeler, David L. Murray, Mindy C. Kohlhagen, Jeffrey A. Vos, Svetlana A. Yatsenko, Mounzer E. Agha, Miroslav Djokic, Steven H. Swerdlow, Nathanael G. Bailey

**Affiliations:** 1 Department of Pathology, UPMC, Pittsburgh, PA USA; 2 Department of Pathology, University of Pittsburgh and UPMC, Pittsburgh, PA USA; 3 Department of Laboratory Medicine and Pathology, Mayo Clinic, Rochester, MN USA; 4 Department of Pathology, Anatomy and Laboratory Medicine, West Virginia University, Morgantown, WV USA; 5 Hillman Cancer Center, University of Pittsburgh and UPMC, Pittsburgh, PA USA

**Correction: Diagnostic Pathology 17, 63 (2022)**.

10.1186/s13000-022-01244-1

Following publication of the original article [1], the authors found a typo in one of the author names in the author group section. Instead of the accurate author name “Jeffrey A. Vos,“ it displays the wrong author name “Jeffrey A. VosUPMC” in the web article that has been published. The original article has been updated.
